# Dexmedetomidine Protects Against Lipopolysaccharide-Induced Acute Kidney Injury by Enhancing Autophagy Through Inhibition of the PI3K/AKT/mTOR Pathway

**DOI:** 10.3389/fphar.2020.00128

**Published:** 2020-02-25

**Authors:** Yuan Zhao, Xiujing Feng, Bei Li, Jichen Sha, Chaoran Wang, Tianyuan Yang, Hailin Cui, Honggang Fan

**Affiliations:** Heilongjiang Key Laboratory for Laboratory Animals and Comparative Medicine, College of Veterinary Medicine, Northeast Agricultural University, Harbin, China

**Keywords:** acute kidney injury, dexmedetomidine, LPS, autophagy, apoptosis

## Abstract

**Background:**

Acute kidney injury (AKI) is often secondary to sepsis. Previous studies suggest that damaged mitochondria and the inhibition of autophagy results in AKI during sepsis, but dexmedetomidine (DEX) alleviates lipopolysaccharide (LPS)-induced AKI. However, it is uncertain whether the renoprotection of DEX is related to autophagy or the clearance of damaged mitochondria in sepsis-induced AKI.

**Methods:**

In this study, AKI was induced in rats by injecting 10 mg/kg of LPS intraperitoneally (i.p.). The rats were also pretreated with DEX (30 μg/kg, i.p.) 30 min before the injection of LPS. The structure and function of kidneys harvested from the rats were evaluated, and the protein levels of autophagy-related proteins, oxidative stress levels, and apoptosis levels were measured. Further, atipamezole (Atip) and 3-Methyladenine (3-MA), which are inhibitors of DEX and autophagy, respectively, were administered before the injection of DEX to examine the protective mechanism of DEX.

**Results:**

Pretreatment with DEX ameliorated kidney structure and function. DEX decreased the levels of blood urea nitrogen (BUN) and creatinine (Cre), urine kidney injury molecule-1 (KIM-1), neutrophil gelatinase-associated lipocalin (NGAL), reactive oxygen species (ROS), and apoptosis proteins (such as cleaved caspase-9 and cleaved caspase-3). However, DEX upregulated the levels of autophagy and mitophagy proteins, such as Beclin-1, LC3 II and PINK1. These results suggest that DEX ameliorated LPS-induced AKI by reducing oxidative stress and apoptosis and enhancing autophagy. To promote autophagy, DEX inhibited the phosphorylation levels of PI3K, AKT, and mTOR. Furthermore, the administration of Atip and 3-MA inhibitors blocked the renoprotection effects of DEX.

**Conclusions:**

Here, we demonstrate a novel mechanism in which DEX protects against LPS-induced AKI. DEX enhances autophagy, which results in the removal of damaged mitochondria and reduces oxidative stress and apoptosis in LPS-induced AKI through the α_2_-AR and inhibition of the PI3K/AKT/mTOR pathway.

## Introduction

Sepsis, a systemic inflammatory response syndrome (SIRS), is caused by infection, can induce multiple organs dysfunction and even death (Bellomo et al., 2017). According to the 2018 World Health Organization report, more than 30 million people worldwide are diagnosed with sepsis each year (Kumar et al., 2019). The mortality rate of patients with sepsis is more than 10%, but increases to 40% in severe cases, such as septic shock (Assinger et al., 2019). The kidney is one of the most vulnerable organs during sepsis, and acute kidney injury (AKI) occurs in 40–50% of septic patients with a mortality rate of up to 60% (Plotnikov et al., 2019). The underlying pathogenesis of sepsis acute kidney injury (SAKI) is not well understood but includes oxidative stress (Chen et al., 2019), inflammation (Feng et al., 2019), and apoptosis (Kang et al., 2018). Recently, it was reported that autophagy played a key role in SAKI, and the inhibition of autophagy resulted in the development of AKI during sepsis (Ho et al., 2016). However, the specific role of autophagy in SAKI remains unclear.

Autophagy is a mechanism which recycles damaged organelles and cellular components in response to various stress conditions such as nutrient deprivation (Filomeni et al., 2015), metabolic balance (Maiuri et al., 2007), apoptosis (Maiuri et al., 2007) and inflammation (Kimura et al., 2017), and is beneficial for cell survival. Clinical and preclinical studies confirm that sepsis triggers autophagy in multiple organs, including the kidney (Wan et al., 2016; Chung et al., 2017; Kimura et al., 2017; Sun et al., 2019), and an increasing amount of evidence suggests that stimulating autophagy *via* pharmacological approaches protects the kidney during sepsis (Mei et al., 2016). Mitophagy, the selective removal of mitochondria by autophagy, also plays an important role in SAKI (Kaushal and Shah, 2016). Mitophagy is necessary to maintain cellular homeostasis and ensure healthy mitochondria. Mitophagy is regulated by two pathways, including the PTEN-induced putative protein kinase 1 (PINK1)/Parkin-dependent and PINK1/Parkin-independent (e.g., BNIP3 and FUNDC1) pathways (Ishimoto and Inagi, 2016). The PINK1/Parkin pathway is the main regulator of mitophagy and has been studied in multiple disease models (Kaushal and Shah, 2016; Sorrentino et al., 2017). Mitophagy can limit cell death from mitochondrial oxidant stress and promote the release of pro-apoptotic proteins (Sun et al., 2018). It has been demonstrated that mitophagy plays a critical role in mitochondrial quality control, tubular cell survival, and renal function during ischemic AKI (Tang et al., 2018). Therefore, autophagy and mitophagy may play key roles in SAKI by reducing oxidant stress and apoptosis.

Dexmedetomidine (DEX) is a highly selective alpha-2 adrenergic receptor (α_2_-AR) agonist (Kawazoe et al., 2017). Our previous studies have shown that DEX exhibits anti-inflammatory and anti-oxidative effects in LPS-induced AKI (Chen et al., 2019; Feng et al., 2019; Yao et al., 2019). It has also demonstrated that DEX has anti-inflammatory and anti-apoptotic effects in LPS-induced AKI (Kang et al., 2018). In the macrophage cell line RAW 264.7, DEX alleviated LPS-induced apoptosis and inflammation through PINK1-mediated mitophagy, which eliminated damaged mitochondria (Wang et al., 2019). Furthermore, it has been shown that DEX alleviated LPS-induced acute lung injury through the PI3K/AKT/mTOR pathway (Meng et al., 2018). Inhibition of the kinase mammalian target of rapamycin (mTOR), a major modulator of autophagy, promotes the initiation of autophagy (Munson and Ganley, 2015). mTOR is a downstream target of the phosphatidylinositol 3 kinase (PI3K) and protein kinase B (AKT) pathway (Heras-Sandoval et al., 2014). AKT activates the mTOR complex 1 (mTORC1) to inhibit autophagy. In this study, we hypothesized that DEX regulates autophagy to alleviate SAKI *via* the PI3K/AKT/mTOR pathway.

Our studies aimed to determine the role of autophagy (including mitophagy) in SAKI, identify the relationship between DEX renoprotection and autophagy during sepsis, and determine how DEX regulates autophagy in SAKI using the autophagy inhibitor 3-Methyladenine (3-MA). Our studies provide a further understanding of the pharmacological mechanism of DEX and increase its clinical application value.

## Materials and Methods

### Animals and Treatment

Adult male Sprague Dawley (SD) rats (weight 180–220 g) were procured from the Second Affiliated Hospital of Harbin Medical University (Harbin, China). Rats were housed under conditions of controlled temperature (21–25°C) and humidity (45–55%), with a 12 h light/dark cycle for 7 days to adjust to the environment. Rats were housed in groups (3 per cage) and received tap water and a standard rat chow diet ad libitum. All experimental procedures in this study adhered the requirements of the Animal Experimental Committee of Northeast Agricultural University and complied with the National Institutes of Health Guide for the Care and Use of Laboratory Animals.

Thirty-six rats were randomly divided into six groups (n = 6) as follow:

1. The CON group did not receive any treatments.
2. The CON+DEX group was injected intraperitoneally (i.p.) with DEX (30 μg/kg, American Pfizer) without any additional treatments.
3. The LPS group received LPS (10 mg/kg, i.p., *Escherichia coli* O111:B4, L2630, Sigma-Aldrich, USA) 4 h before sacrifice.
4. The DEX+LPS group was injected with DEX 30 min before the injection of LPS.
5. The Atip+DEX+LPS group was injected with atipamezole (Atip) (an α_2_-AR antagonist) (300 mg/kg, i.p., American Pfizer) 30 min before injection of DEX; 30 min after the injection of DEX, injection of LPS.
6. The 3-MA+DEX+LPS group was injected with 3-MA (30 mg/kg, i.p., S1075, Selleck, Shanghai, China) 30 min before injection of DEX; 30 min after the injection of DEX, injection of LPS.

Four hours after the last treatment, all rats were quickly anesthetized with isoflurane (Yipin Pharmaceutical Co., Ltd., Hebei, China) to collect blood, urine, and kidney samples and no rats died until sacrificed.

### Assessment of Kidney Function

Blood samples were collected quickly by heart puncture. After coagulating 30 min, the blood samples were centrifuged at 3,000 rpm for 10 min at room temperature to collect serum. Using an automated analyzer (Roche Diagnostics, Mannheim, Germany) detected Serum blood urea nitrogen (BUN) and creatinine (Cre) levels. Using enzyme-linked immunosorbent assay analyzed kidney injury molecule-1 (KIM-1) (RKM100, R&D Systems, USA) and neutrophil gelatinase-associated lipocalin (NGAL) (DY3508, R&D Systems, USA) levels of urine samples.

### ELISA Assay for Serum Inflammation Cytokine

Using a commercial ELISA kit (Nanjing Jiancheng Bioengineering Institute, Nanjing, China) examined serum tumor necrosis factor-α (TNF-α) (H052), interleukin (IL)-1β (H002), IL-6 (H007), and IL-18 (H0015) according to the manufacturer’s instructions.

### Histological and Ultrastructural Observations

Kidney fixed in 10% neutral-buffered formalin solution were dehydrated, seeded, dipped in wax, embedded, sectioned and stained with hematoxylin and eosin stain and then observed by light microscopy (BX-FM; Olympus Corp, Tokyo, Japan). Renal tissue with the following histopathological changes were judged injured: loss of brush border, vacuolization, cast formation, tubular dilation and disruption, cell lysis, and cellular necrosis. Tissue damage was checked in a blinded manner and scored by the percentage of damaged tubules: 0, no damage; 1, 0–25%; 2, 25–50%; 3, 50–75%; 4, more than 75% (Tang et al., 2018).

The kidneys were fixed overnight with 3% glutaraldehyde, rinsed three times with 0.1 M phosphate buffer saline (PBS) for 15 min each time. The pellet was fixed with 1% osmic acid 90 min and then rinsed three times with 0.1 M PBS for 15 min each. Thereafter, the samples were dehydrated in different gradients of ethanol (50, 70, 90, and 100%) for 10 min. Then placed in fresh pure Epon resin and polymerized at 60°C for 2 h. Finally, the ultrathin sections (60 nm) were doubled stained with uranyl acetate and lead citrate. The observation was done on a transmission electron microscope (Tecnai, Hitachi, Tokyo, Japan) at 100 kV Electron Microscopy Film 4489 (Kodak, ESTAR thick base, San Francisco, CA)

### Measurement of Oxidative Stress Markers

According to the manufacturer’s instructions, using the corresponding assay kit (Nanjing Jiancheng Bioengineering Institute, Nanjing, China) measured the levels of reactive oxygen species (ROS) (E004-1-1), glutathione (GSH) (A006-1-1), and malondialdehyde (MDA) (A003-2-2) and tested the activity of superoxide dismutase (SOD) (A001-3-2) and catalase (CAT) (A007-1-1).

### Western Blot Analysis

Equal amounts of protein samples were loaded onto sodium dodecyl sulfate-polyacrylamide gel, electrophoresed, and then transferred to polyvinylidene difluoride membranes. Membranes were blocked in 5% bull serum albumin or skim milk for 2 h at room temperature, incubated with the primary antibody in the anti-dilution buffer (Leagene Biotechnology, Beijing, China) overnight at 4°C. Primary antibodies and dilutions were as follows: Bax (1:500, sc-23959), Parkin (1:500, sc-32282), PINK1 (1:500, sc-517353), caspase-3 (1:500, sc-373730), caspase-9 (1:500, sc-56076), p62 (1:500, sc-48402) and p-AKT (1:500, sc-514032) from Santa Cruz Biotechnology, USA. Cytochrome C (Cyt C) (1:1000, WL02410); PI3 Kinase p85 (1:500, WL02240), GAPDH (1:2,000, WL01547), p-mTOR (1:500, WL03694), AKT (1:1,000, WL0003b) and Bcl-2 (1:500, WL01556) from Wanlei Bio, China. PI3 Kinase p85 (phospho-Tyr467/199) (1:750, #11508) from Signalway Antibody, USA. MAP1LC3B (1:1,000, A11280) from ABclonal, China. mTOR(1:1,000, A5866) from bimake, USA. Beclin-1(1:1,000, #3495) from Cell Signaling Technology, USA. After washed with Tris-buffered saline containing Tween (TBST), the membranes were incubated with horseradish peroxidase (HRP)-conjugated goat anti-rabbit IgG antibody (1:20,000, ZB-2301, ZSGB-Bio, Beijing, China) or anti-mouse IgG antibody (1:20,000, ZB-2305, ZSGB-Bio, Beijing, China) for 2 h at room temperature then washed with TBST. Protein bands were visualized using ECL plus Western Blot Detection Reagents (GE Healthcare).

### Analysis of Apoptosis

Using TUNEL Apoptosis Assay kit (11684795910, Roche, Basel, Switzerland) measured apoptosis, according to the manufacturer’s instructions. Then samples were examined under a fluorescence microscope after anti fluorescence quenching.

### Real-time Polymerase Chain Reaction (RT-PCR) Analyses

According to the manufacturer’s instructions, Using Eastep Super Total RNA Extraction Kit (Promega Biotech Co, Ltd, Beijing, China) extracted total RNA from kidney. Using NanoDrop2000 (Thermo Fisher Inc., Waltham, MA, USA) tested the purity and concentration of total RNA.

Then according to the manufacturer’s instructions, using GoScript™ Reverse Transcription System (Promega Biotech Co, Ltd, Beijing, China) reversely transcribed total RNA into cDNA. **Table 1** shows the PCR primer sequences. Real-time PCR was implemented in the Roche 480 System (Roche, Switzerland) using SYBR Green PCR Core Reagents (Roche, Switzerland). GAPDH was used as the internal reference for relative quantitative analysis of gene mRNA expression level. Data were analyzed according to the 2^−∆∆Ct^ method.

**Table 1:** Primer sequence in this study.

Gene	No.	Sequences
PINK1	NM_001106694.1	Forward: 5′GTATGAAGCCACCATGCCCA3′ Reverse: 5′ACGACATCTGGGCCTTTTCC3′
MAPLC3b	NM_022867.2	Forward: 5′AGCACTGGCTGTGTAAGACT3′ Reverse: 5′TGCCTACGTTCTGATCTGTGG3′
Beclin-1 (Becn1)	NM_001034117.1	Forward: 5′GATGGAGCAGAAGCCGACTC3′ Reverse: 5′TCGTGTCCAGTTTCAGAGGC3′
p62 (SQSTM1)	NM_175843.4	Forward:5′TGGAGTCGGATAACTGCTCAGGAG3′ Reverse:5′AGACTGGAGTTCACCTGTGGATGG3′
GADPH	NM_017008.4	Forward: 5′AGTGCCAGCCTCGTCTCATA3′ Reverse: 5′GATGGTGATGGGTTTCCCGT3′

### Immunohistochemistry (IHC) and Immunofluorescence (IF) Staining

Expression of Beclin-1, LC3, p62 and Cyt C in kidney tissues was detected by IHC staining. Paraffin-embedded kidney tissue fraction (5 μm thickness) and xylene dewaxed, then dehydrated using graded concentrations of alcohol, and incubation with 3% H_2_O_2_ inhibited endogenous peroxidase. After blocked in 10% goat serum for 10 min at room temperature, incubated with the primary antibody in blocking solution overnight at 4°C. The primary antibodies for IHC were p62 (1:50, sc-48402), Beclin-1 (1:200, WL02508, Wanlei Bio), MAPLC3B (1:50, A11280), and Cyt C (1:200, WL02410). After slides were washed in PBS and applied HRP-labeled anti-rabbit IgG (1:100; Beyotime, China) for 30 min at 37°C, then washed with PBS again. Staining was visualized by reaction with 3,3'-diaminobenzidine (DAB) (Solarbio, Beijing, China) and counterstaining with hematoxylin. Sections were made transparent with xylene. Finally, using the DP73 type microscope (Olympus, Japan) observed sections. We also performed IF co-staining using PINK1 (1:50, sc-517353) and VDAC1 (1:50, sc-390996, Santa Cruz Biotechnology, USA) antibodies to delineate the mitophagy in the kidney. Then images were acquired with a Nikon Eclipse Ni inverted microscope (TE2000, Nikon, Tokyo, Japan).

### Statistical Analysis

Data were expressed as mean ± SEM (standard error means). The data were analyzed by SPSS 18.0 (Chicago, IL, USA). A one-way analysis of variance (ANOVA) followed by Tukey’s *post hoc* test was used to multi-group comparison. Using GraphPad Prism 7 software (GraphPad Software Inc., San Diego, CA, United States) made graphs. Statistically significant were considered to be significant when p < 0.05, and extremely significant when p < 0.01.

## Results

### Effects of DEX on Renal Function and Structural Changes

**Figure 1:**
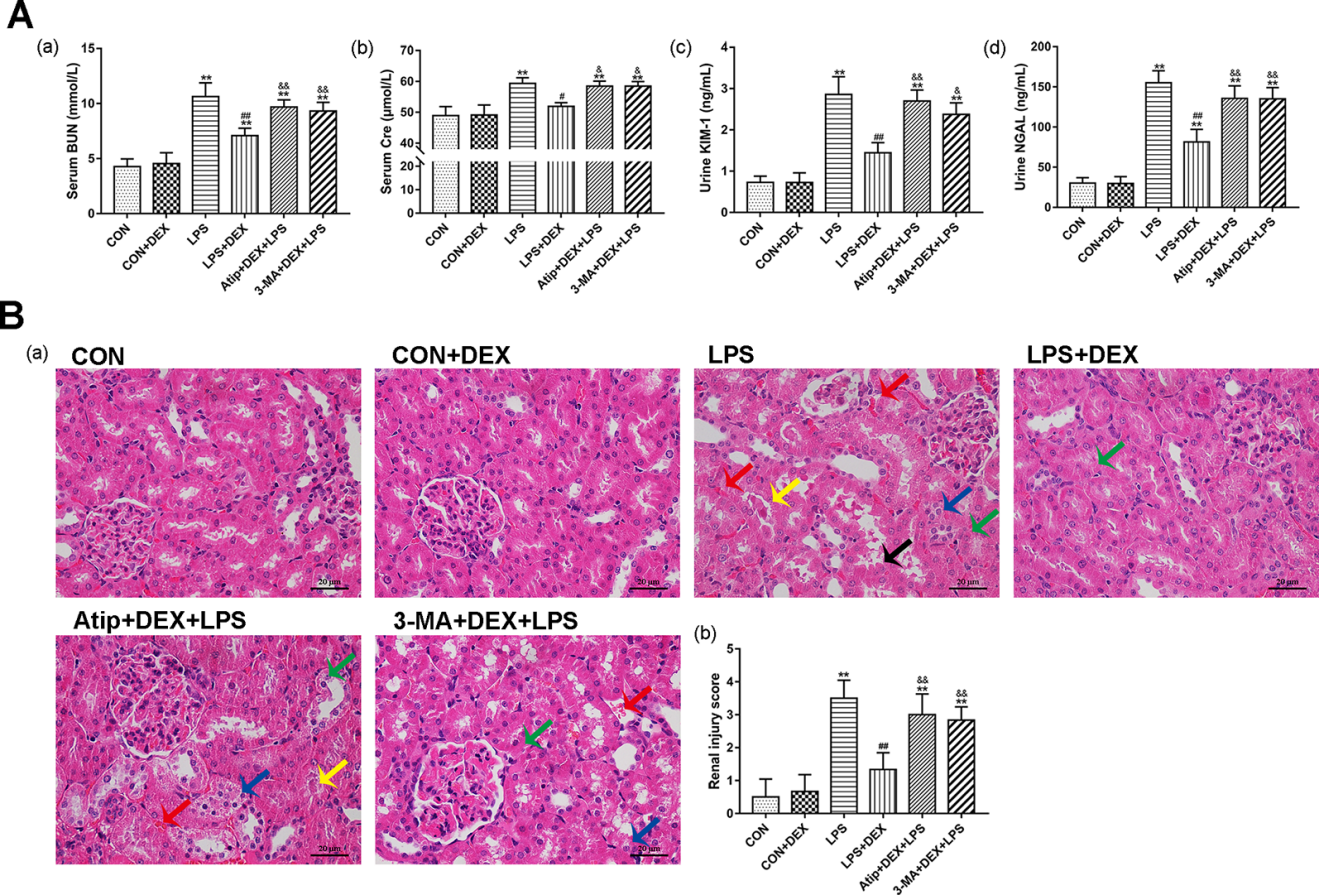
Protective effect of DEX in LPS-induced AKI. **(A)** DEX improved renal dysfunction in LPS-induced AKI: the levels of serum BUN (a), serum Cre (b), urine KIM-1 (c), and urine NGAL (d) in rats. **(B)** Histopathological examination of the kidney: (a) Representative images from different groups. Green arrow indicates edema of renal tubular epithelial cells; blue arrow indicates tubular necrosis; yellow arrow indicates cast formation; red arrow indicates hemorrhage. Magnification: 400×, Bar: 20 μm. (b) Semiquantitative histopathological score of injury. Data are expressed as mean ± SEM (n = 6). **p < 0.01 versus CON group. #p < 0.05, ##p < 0.01 versus LPS group. &p < 0.05, &&p < 0.01 versus DEX + LPS group.

BUN and Cre are important indicators of the severity of renal impairment. Furthermore, NGAL and KIM-1 have been identified as specific biomarkers of kidney injury (Han et al., 2002; Bolignano et al., 2008), and their increased expression is associated with early renal tubular injury in AKI (Chen et al., 2019). The levels of BUN (**Figure 1Aa**), Cre (**Figure 1Ab**), KIM-1 (**Figure 1Ac**), and NGAL (**Figure 1Ad**) were significantly increased after LPS injection, and DEX pretreatment significantly decreased the levels of these markers, which indicated that DEX alleviates LPS-induced kidney injury. However, the protective effects of DEX were reversed by treatment with Atip and 3-MA.

Analysis of the pathological sections of rat kidneys revealed intact glomerular and tubular structures in the CON group (**Figure 1B**). In contrast, there were substantial pathological changes in the LPS group, which included edema of renal tubular epithelial cells (green arrow), tubular necrosis (blue arrow), telangiectasia and severe congestion/hemorrhage (red arrow), and cast formation (yellow arrow) (**Figure 1B**). However, the LPS-induced kidney damage was significantly improved by pretreatment with DEX. In addition, the protective effect of DEX was reversed by Atip and 3-MA, and similar damage was observed in these two groups as in the LPS group.

**Figure 2:**
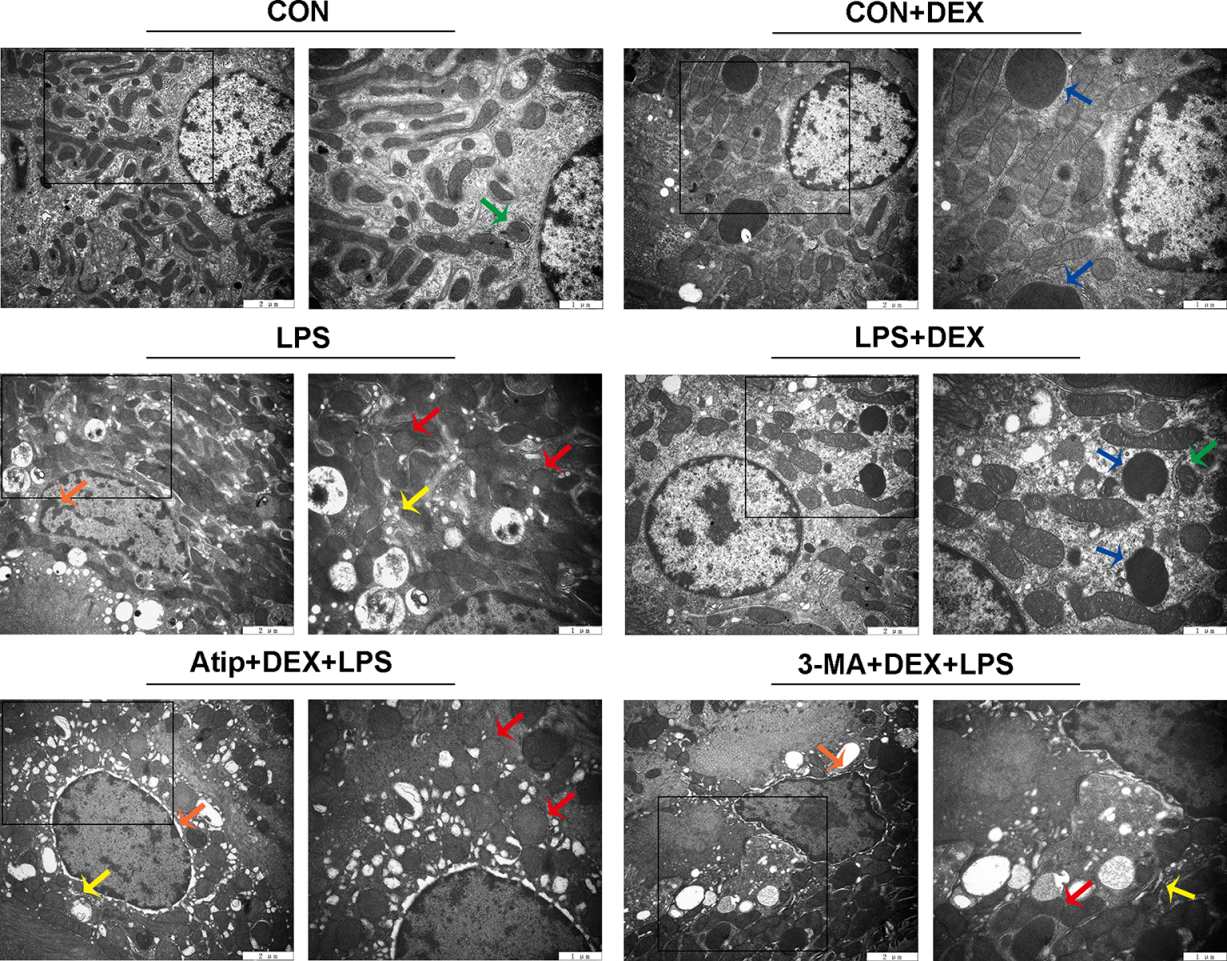
The ultrastructure examination of the kidney. Representative ultrastructure samples from different groups. Red arrow indicates mitochondria appeared swelling, vacuolization and loss of cristae; orange arrow indicates chromatin margination, condensation, and fragmentation; yellow arrow indicates endoplasmic reticulum swelling; blue arrow indicates autolysosome; green arrow indicates mitophagy.

Renal ultrastructure changes are shown in **Figure 2**. In the CON group, cells had an intact nuclear morphology, and cell mitochondria exhibited normal morphology with clearly discernible cristae. In the LPS group, we observed mitochondria defects, such as swelling, disorganization, and reduction or vanishing of the cristae (red arrow). We also observed typical apoptotic features, including chromatin margination, condensation, and fragmentation (orange arrow), in the LPS group. These results suggest damaged mitochondria induced apoptosis in LPS-induced AKI. DEX pretreatment alleviated cell mitochondria injury and apoptosis that were induced by LPS, and we also observed mitophagy (green arrow) and autolysosomes (blue arrow). However, the administration of Atip and 3-MA reversed the effects of DEX. These results suggest that DEX alleviates LPS-induced kidney injury *via* the α_2_-AR and the enhancement of autophagy.

### Effects of DEX on Renal Inflammation and Oxidative Stress

**Figure 3:**
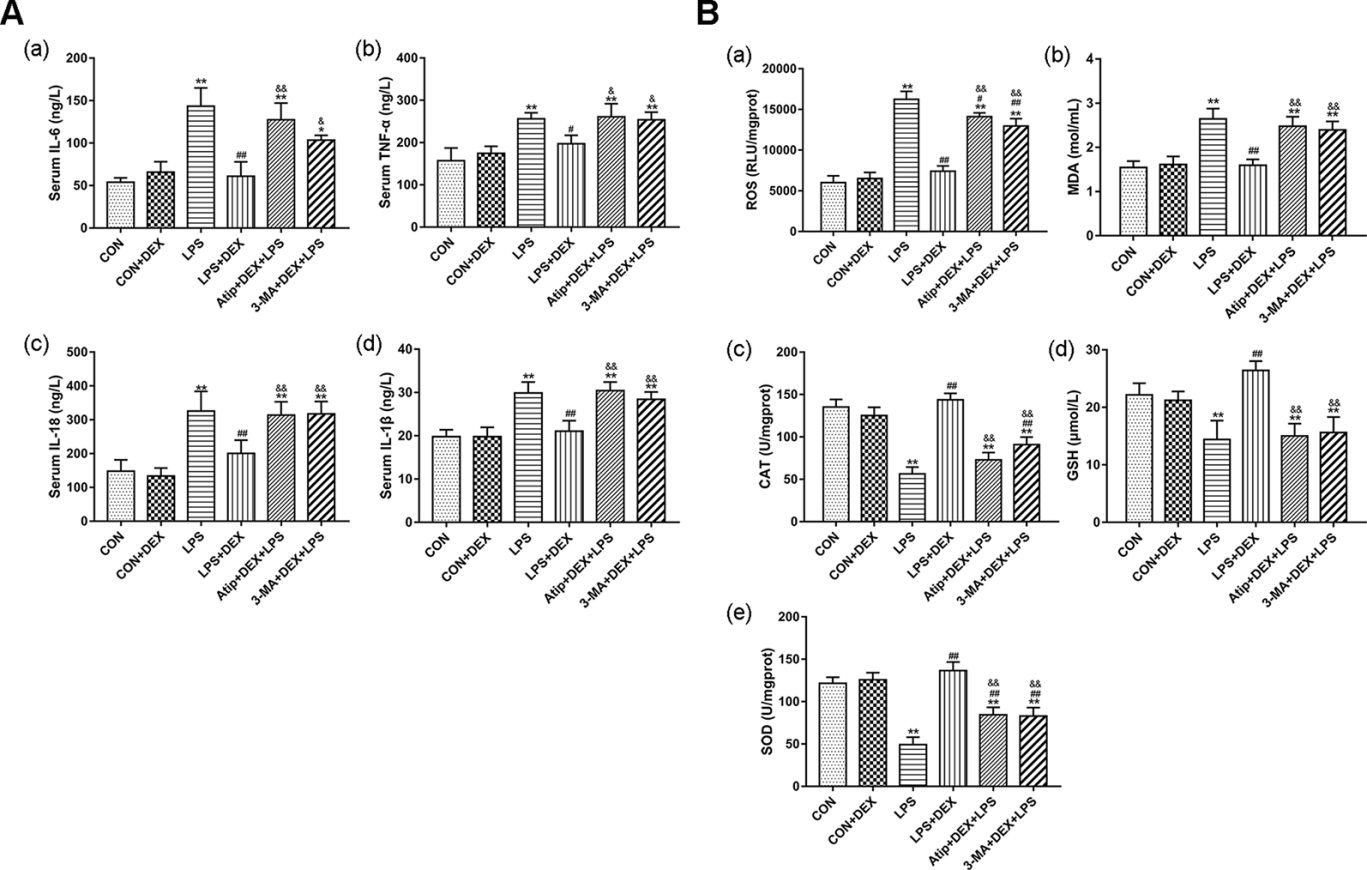
DEX reduced inflammation and oxidative stress in LPS-induced AKI. **(A)** The content of inflammatory cytokines in serum, including IL-6 (a), TNF-α (b), IL-18 (c), IL-1β (d). **(B)** The content of ROS (a); the concentration of MDA (b); the activity of CAT (c); the content of GSH (d); the activity of SOD (e). Data are expressed as mean ± SEM (n = 6). *p < 0.05, **p < 0.01 versus CON group. #p < 0.5, ##p < 0.01 versus LPS group. &p < 0.05, &&p < 0.01 versus DEX + LPS group.

To further elucidate the relationship between DEX renoprotection and autophagy, we measured the levels of inflammatory cytokines and oxidative stress. LPS significantly elevated the levels of interleukin (IL)-6 (**Figure 3Aa**), tumor necrosis factor-alpha (TNF-α) (**Figure 3Ab**), IL-18 (**Figure 3Ac**), and IL-1β (**Figure 3Ad**), whereas DEX significantly decreased the levels of these four markers. Compared with the LPS+DEX group, the levels of these inflammatory cytokines were significantly increased in the Atip +DEX+LPS and 3-MA+DEX+LPS groups. These results suggest that DEX reduces the levels of inflammatory cytokines *via* the α_2_-AR and the enhancement of autophagy.

We measured the levels of ROS, CAT, MDA, SOD, and GSH in kidney tissues. In the LPS group, the levels of ROS (**Figure 3Ba**) and MDA (**Figure 3Bb**) were significantly elevated, and the levels of CAT (**Figure 3Bc**), GSH (**Figure 3Bd**), and SOD (**Figure 3Be**) were markedly decreased. DEX not only significantly reduced ROS and MDA levels, but also increased SOD and CAT activity and increased GSH levels. However, 3-MA and Atip significantly reversed these DEX-induced changes, suggesting that DEX protects against oxidative stress *via* the α_2_-AR and the enhancement of autophagy.

### Effects of DEX on Renal Apoptosis

**Figure 4:**
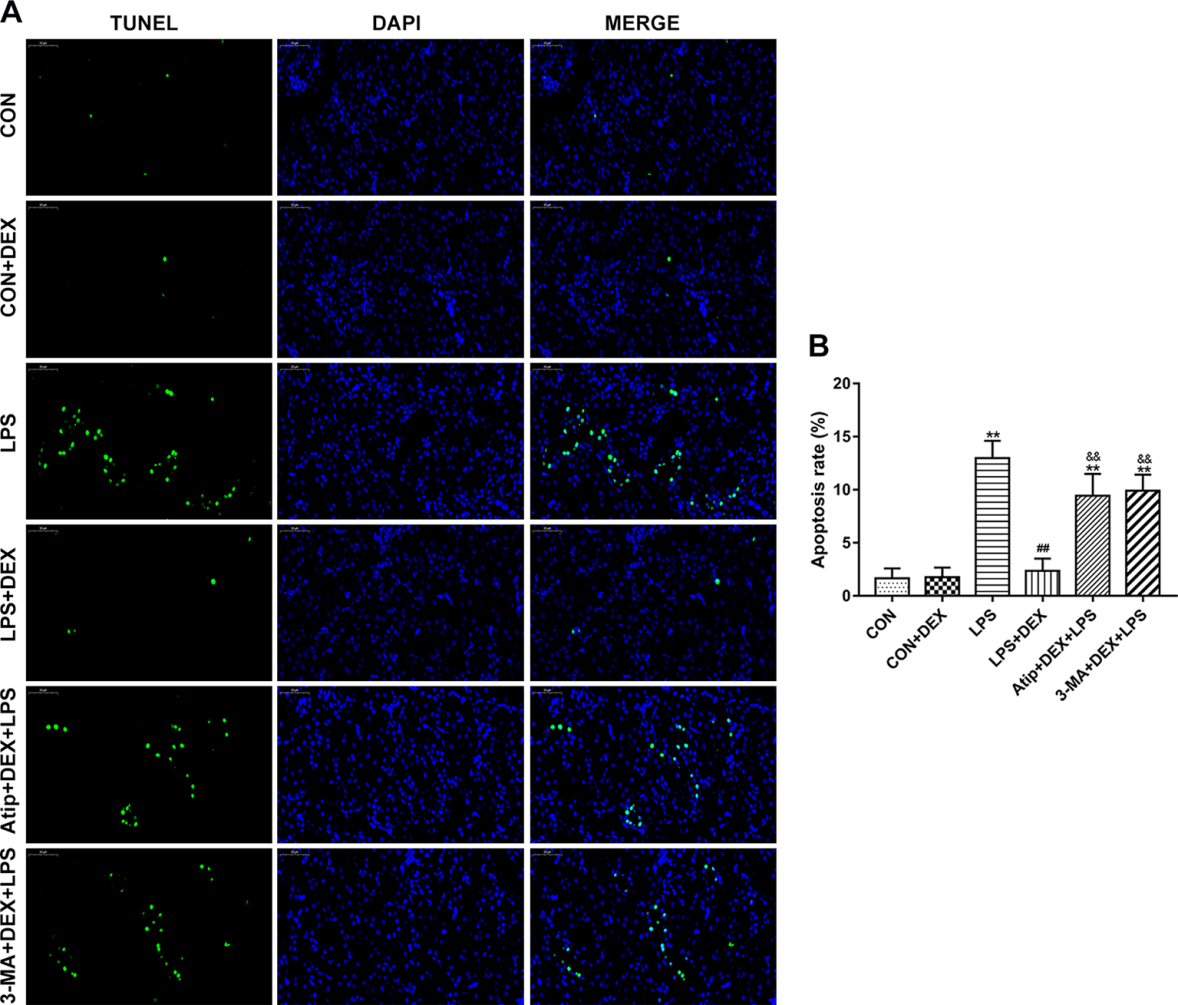
Effect of DEX on renal apoptosis. **(A)** Representative images of TUNEL assay in renal tissue. Bar: 50 μm. **(B)** TUNEL-positive cells rate in kidney tissues. Data are expressed as mean ± SEM (n = 6). **p < 0.01 versus CON group. ##p < 0.01 versus LPS group. &&p < 0.01 versus DEX + LPS group.

Using the terminal uridine nick-end labeling (TUNEL) assay, we measured apoptosis in kidney tissue sections. There were a few apoptotic cells in the CON group, but apoptosis was elevated in the LPS group. However, the levels of apoptosis were decreased in the DEX group compared with the LPS group, and the levels of apoptosis were significantly increased in the Atip+DEX+LPS and 3-MA+DEX+LPS groups compared with the LPS+DEX group (**Figures 4A, B**).

**Figure 5:**
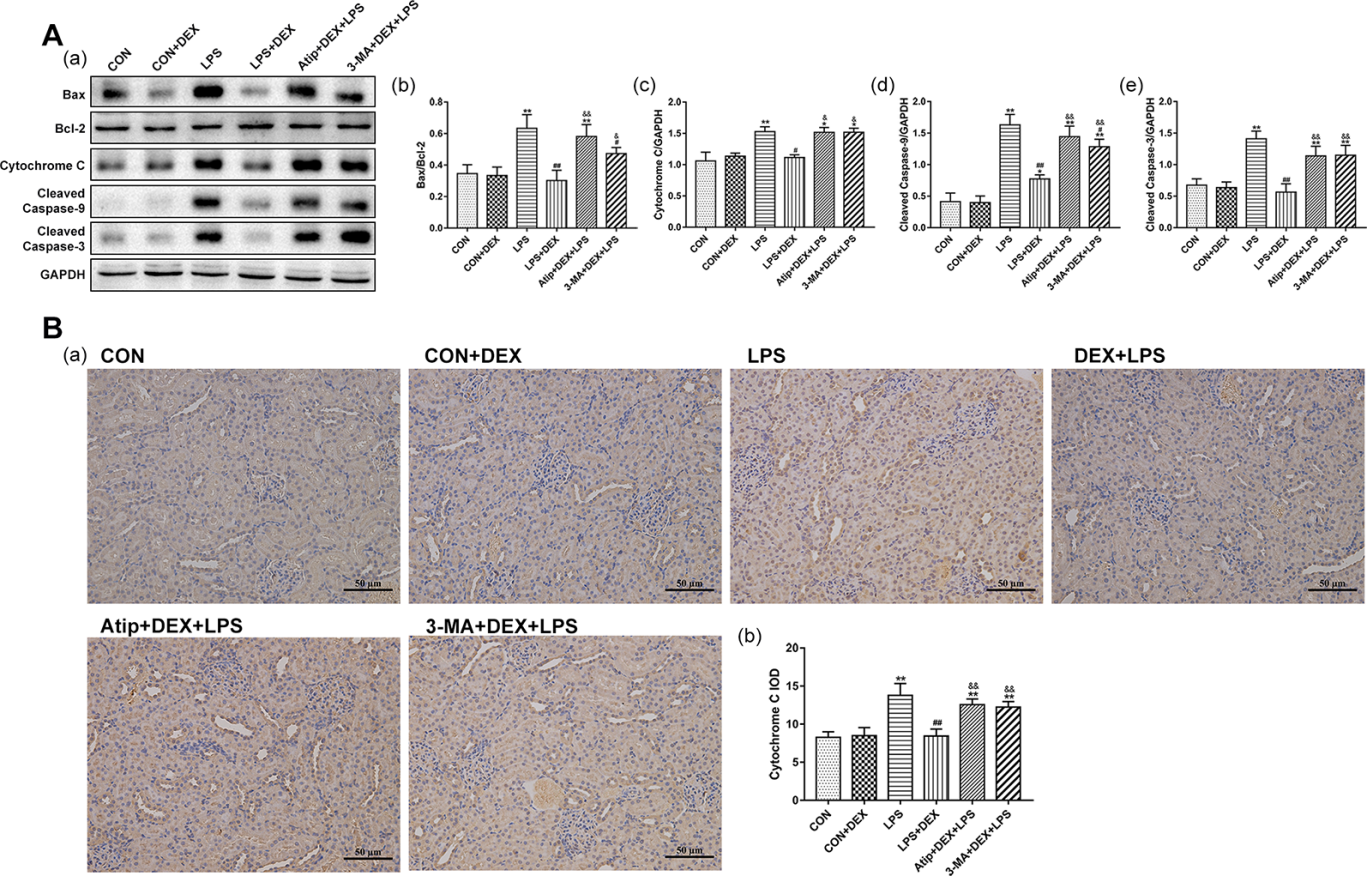
Effects of DEX on apoptosis related proteins. **(A)** Representative blots (a) of Bax, Bcl-2, Cyt C, cleaved caspase-9, and cleaved caspase-3, and the densitometry of Bax/Bcl-2 (b), Cyt C (c), cleaved caspase-9 (d), and cleaved caspase-3 (e) signals. **(B)** Representative images of Cyt C IHC (a), and quantitative analysis of Cyt C (b) in kidney. Data are expressed as mean ± SEM (n = 6). **p < 0.01 versus CON group. #p < 0.05, ##p < 0.01 versus LPS group. &p < 0.05, &&p < 0.01 versus DEX + LPS group.

We also determined the expression of apoptosis-related proteins and the ratio of Bax/Bcl-2 (**Figure 5Ab**). The levels of apoptosis-related proteins, such as Bax (**Figure 5Aa**), Cyt C (**Figures 5Aa, c, B**), cleaved caspase-9 (**Figure 5Aa, d**), and cleaved caspase-3 (**Figure 5Aa, e**), were significantly elevated in the LPS group. However, DEX reduced the levels of these apoptosis-related proteins. Additionally, the administration of Atip and 3-MA reversed the effect of DEX. These results indicate that LPS induces mitochondrial apoptosis in AKI, and DEX reduces cell apoptosis *via* the α_2_-AR and the enhancement of autophagy.

### Effect of DEX on Renal Autophagy and the PINK1/Parkin Mitophagy

**Figure 6:**
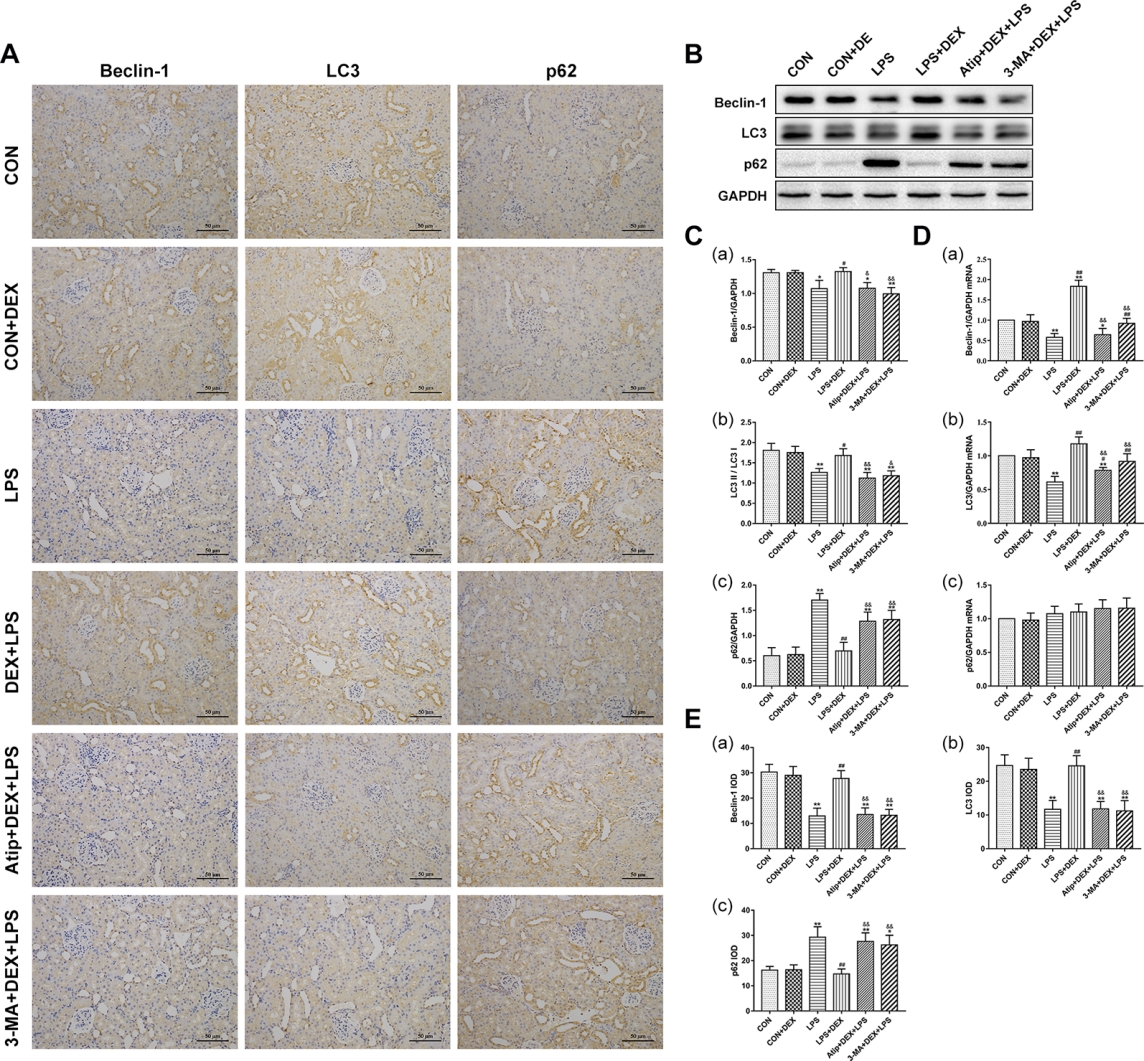
Effects of DEX treatment on autophagy. **(A)** Representative images of Beclin-1 IHC, LC3 IHC, and p62 IHC. **(B)** Representative blots of Beclin-1, LC3, and p62. **(C)** Representative blots densitometry of Beclin-1 (a), LC3 (b), and p62 (c). **(D)** The expression of Beclin-1 (a), LC3 (b), and p62 (c)mRNA in renal tissues. **(E)** The quantitative analysis of Beclin-1 IHC (a), LC3 IHC (b), and p62 IHC (c) in kidney. Data are expressed as mean ± SEM (n = 6). *p < 0.05, **p < 0.01 versus CON group. #p < 0.05, ##p < 0.01 versus LPS group. &p < 0.05, &&p < 0.01 versus DEX + LPS group.

Beclin-1 is a key molecule that controls autophagic activity. LC3 is a marker of autophagosome formation, and the ratio of LC3 II/LC3 I correlates to the number of autophagosomes. The levels of Beclin-1 (**Figures 6A–C, E**) and LC3 II (**Figures 6A–C, E**) were decreased in the LPS group but increased after pretreatment with DEX. The polyubiquitin-binding protein p62/SQSTM1 (**Figures 6A–C, E**), which degraded during autophagy, showed the reverse trend. Treatment with Atip and 3-MA reversed the effect of DEX. Similar changes in the mRNA and protein of Beclin-1 and LC3 were observed (**Figures 6Da, b**), but p62 mRNA did not change in every group (**Figure 6Dc**). These results indicate that LPS inhibits autophagy, and DEX enhances autophagy *via* the α_2_-AR.

**Figure 7:**
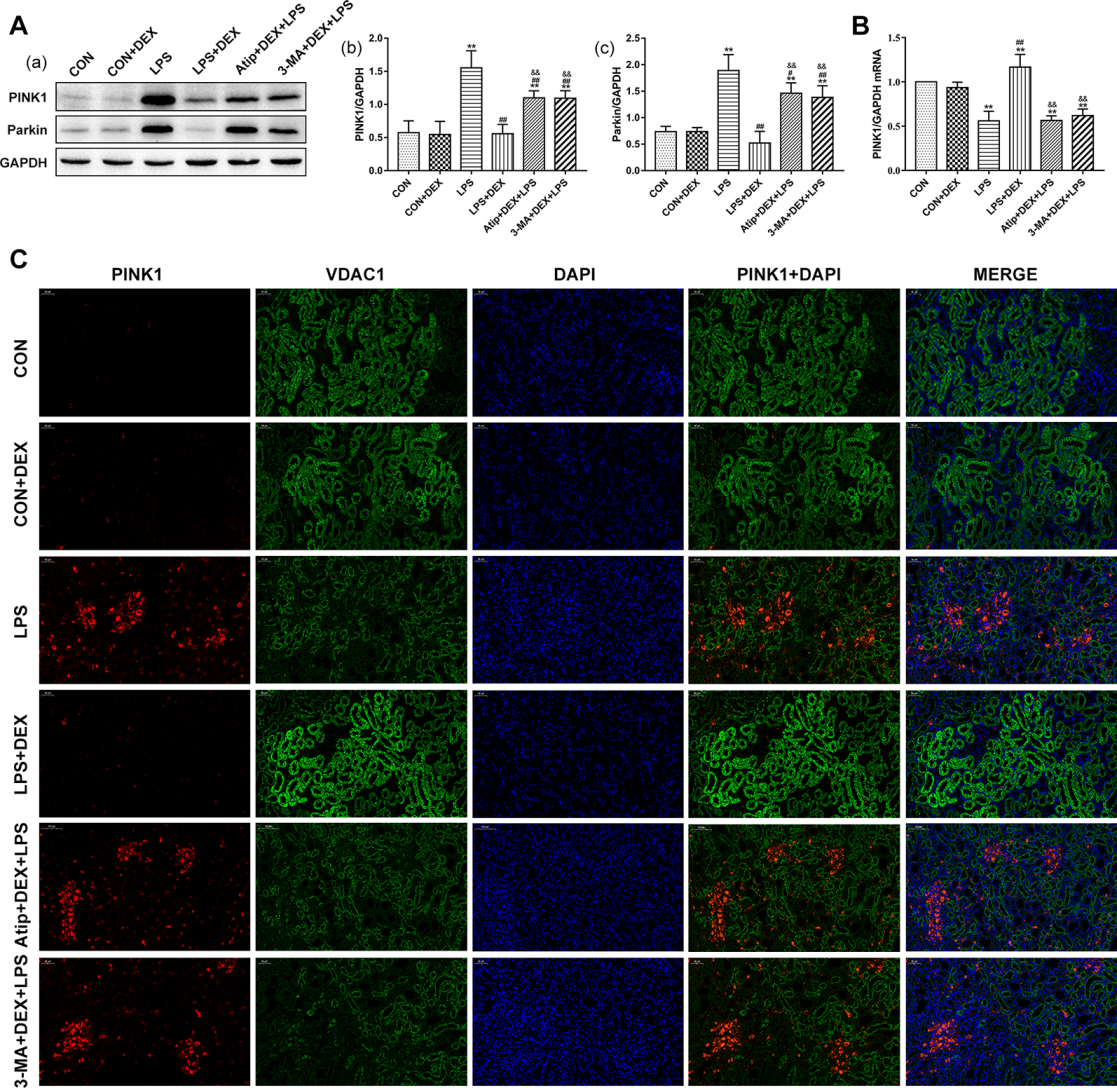
Effects of DEX treatment on mitophagy. **(A)** Representative blots (a) and densitometry of PINK1 (b) and Parkin (c) signals. **(B)** The expression of PINK1 mRNA in renal tissue. **(C)** Representative images of PINK1 localization in renal tissues. Bar: 50 μm. Positive PINK1 cells were stained red, positive VDAC1 cells were stained green, with the sections counterstained with DAPI to visualize nuclei. Data are expressed as mean ± SEM (n = 6). **p < 0.01 versus CON group. #p < 0.05, ##p < 0.01 versus LPS group. &&p < 0.01 versus DEX + LPS group.

Having observed mitophagy (**Figure 2**) from renal ultrastructure changes in the LPS+DEX group, we next measured the levels of mitophagy-related proteins. The amount of PINK1 was very low in the CON group. PINK1 largely accumulated on mitochondria in the LPS group, but the pretreatment with DEX decreased the amount of PINK1 (**Figures 7A, C**). The changes in Parkin (**Figure 7A**) were consistent with PINK1. The change in PINK mRNA (**Figure 7B**) was a reverse trend compared with the change of protein, which suggested LPS downregulated the transcription of PINK1, whereas DEX upregulated its transcription. Further, the use of Atip and 3-MA reversed the effects of DEX.

### DEX Regulates Autophagy *via* Inhibiting the PI3K/AKT/mTOR Pathway

**Figure 8:**
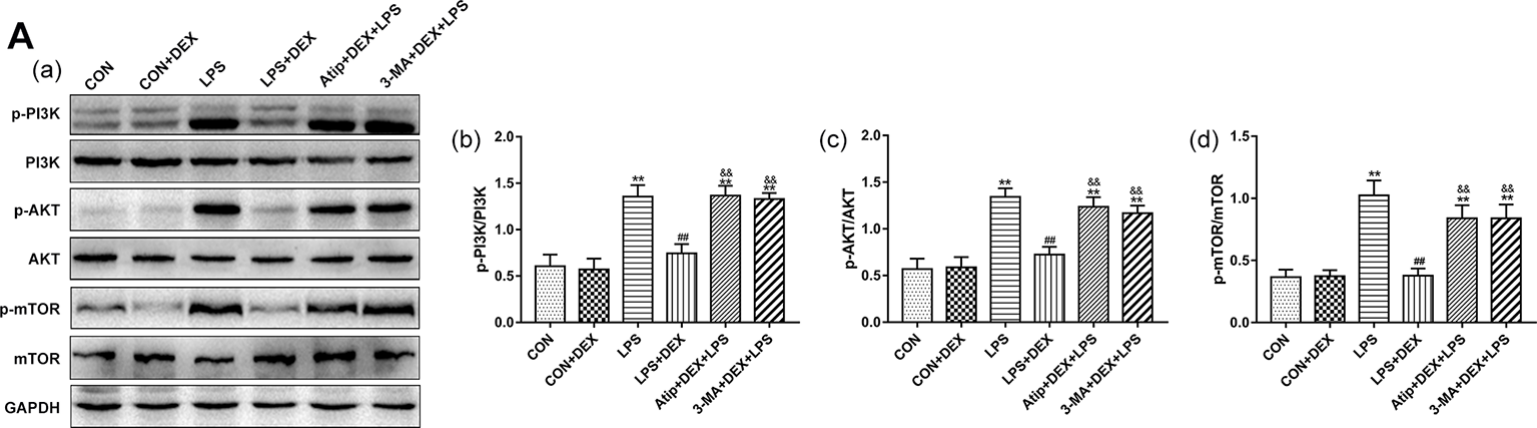
Effects of DEX treatment on the PI3K/AKT/mTOR pathway. **(A)** Representative blots (a), and densitometry of p-PI3K/PI3K (b), p-AKT/AKT (b), and p-mTOR/mTOR (c) signals. Data are expressed as mean ± SEM (n = 6). **p < 0.01 versus CON group. ##p < 0.01 versus LPS group. &&p < 0.01 versus DEX + LPS group.

The mechanism underlying the protective effect of DEX through the modulation of autophagy was further investigated by assessing the levels of PI3K, AKT, and mTOR in kidney tissues (**Figure 8A**). In the LPS group, the phosphorylation levels of PI3K, AKT, and mTOR were increased compared with the CON group. However, pretreatment with DEX reduced these protein phosphorylation levels compared with the LPS group, and Atip reversed the effects of DEX. These results indicate that DEX inhibited the PI3K/AKT/mTOR pathway *via* the α_2_-AR.

## Discussion

In our previous study, we demonstrated that DEX plays a protective role against SAKI *via* attenuating oxidative stress and downregulating inflammatory factors. Here, we further examined the mechanisms underlying the protective effect of DEX on SAKI by focusing on the potential involvement of DEX in the modulation of autophagy.

LPS, a known toxic component of Gram-negative bacteria, is widely used to induce SAKI in animal models (Jou-Valencia et al., 2018), and studies have confirmed that the intraperitoneal administration of LPS for 4 h induces AKI (Chen et al., 2019; Feng et al., 2019). After LPS treatment, the indicators of kidney injury (BUN, Cre, KIM-1, and NGAL) are significantly increased, suggesting renal dysfunction and kidney injury. Additionally, the integrity of glomerular and tubular structures is disrupted, and edema of renal tubular epithelial cells, tubular necrosis, and cast formation are observed. In renal tissue sections, telangiectasia, severe congestion, and hemorrhage also occur. These structural changes indicate that LPS destroys renal tissue structure and induces AKI.

Our results show that oxidative stress, inflammation, and apoptosis occur in the kidney after the administration of LPS. Meanwhile, the inhibition of autophagy occurs in the kidney. We observed the reduction of Beclin-1 and LC3 II and the accumulation of p62 in the kidney after treatment with LPS. However, another study showed that renal autophagy was rapidly induced by LPS, and this was followed by the progression from autophagosome to autolysosome, and finally by autophagic degradation (Mei et al., 2016).These results are different from ours, but differences in autophagy exist in many studies, and these differences are currently being studied by many research groups. These differences are likely associated with the use of various experimental models and the time-points monitored. Recently, Yuxiao Sun confirmed that in a mouse heart, autophagy increased in a dose-dependent manner at low doses of LPS (0.25–2.5 mg/kg), but this was followed by a gradual decline at higher doses of 5–15 mg/kg (Sun et al., 2018). This suggests that autophagy was upregulated in less severe or mild sepsis but inhibited during severe sepsis. Our results also show that the inhibition of autophagy occurred in LPS-induced AKI, and our model in which 10 mg/kg of LPS was administered for 4 h can also represent severe sepsis in rats.

Oxidative stress and apoptosis are known as the main pathological mechanisms of AKI. From the analysis of GSH, SOD, CAT, MAD, and ROS, it is clear that redox imbalance occurs in the kidney during sepsis. A large number of ROS triggers changes in mitochondrial structure and impairs mitochondrial function. As a result of the damage, mitochondria, the main source of ROS, produce more ROS (Lowes et al., 2013), which causes the body to enter a vicious cycle which aggravates kidney damage. At the same time, the damaged mitochondria also release mitochondrial-derived danger-associated molecular patterns (DAMPs), including Cyt C, which induces apoptosis (Sun et al., 2018).After the administration of LPS, mitochondria in kidney tubular cells were damaged and exhibited swelling, fragmentation, vacuoles in the mitochondrial matrix, and loss of cristae. Upon mitochondrial damage, Bax translocates from the cytosol to the mitochondria, increasing mitochondrial permeability and opening the mitochondrial permeability transition pore (MPTP). Concomitantly, Cyt C is released from mitochondria to the cytosol (Wei et al., 2001),which stimulates Apaf-1 (apoptotic protease-activating factor 1) to activate caspase-9. Once activated, caspase-9 activates downstream effector caspases, such as caspase-3, to promote apoptosis (Adams and Cory, 2007). The kidney is second only to the heart in its abundance of mitochondria and consumption of oxygen (Parikh et al., 2015; Kaushal and Shah, 2016). Mitochondria provide high energy for the processes of the excretion of waste products and electrolyte reabsorption. Therefore, for protection against LPS-induced AKI, abnormal mitochondria must be removed to maintain the quality control of mitochondria.

Autophagy can non-selectively or selectively eliminate damaged proteins and organelles by forming a double-membrane autophagosome, which fuses with lysosomes (Wu et al., 2019). Mitophagy is the selective autophagy of mitochondria. Under basal conditions, PINK1 targets mitochondria and is imported and degraded by healthy mitochondria (Whitworth and Pallanck, 2017), and PINK expression is also very low and not easily detected. Under stress conditions, PINK1 senses mitochondrial damage and activates Parkin by phosphorylating Parkin and ubiquitin. Activated Parkin then builds ubiquitin chains on damaged mitochondria to tag them for degradation in lysosomes (Bingol and Sheng, 2016).In this study, PINK1 largely accumulated on mitochondria in LPS-induced AKI, indicating LPS-induced abnormal mitochondria were recognized by PINK1. We also observed the reduction of LC3II and Beclin-1 and the accumulation of p62. It is well established that Beclin-1, p62, and LC3 are the key proteins of autophagy. Beclin-1 interacts with the class III phosphatidylinositol 3-kinase (PtdIns3K) to initiate autophagy and participates in later steps involving the fusion of autophagosomes to lysosomes (Zhong et al., 2009). LC3 binds to autophagosome membranes to form a complete autophagosome that fuses with lysosomes for degradation (Wu et al., 2019). P62 interacts with LC3 II to target aggregates for autophagy-specific degradation (Parzych and Klionsky, 2014). Therefore, our results suggested that at the late stages of mitophagy, complete autophagosomes could not form and fuse with lysosomes for degradation, which is consistent with the inhibition of autophagy. Finally, damaged mitochondria were recognized by PINK1, but not degraded through autophagy, which allowed them to accumulate in the cell. These factors aggravated kidney damage. Therefore, the occurrence and development of LPS-induced AKI are associated with the inhibition of autophagy, and enhancing autophagy to remove abnormal mitochondria may be an important mechanism to ameliorate LPS-induced AKI.

We have confirmed that DEX protects against LPS-induced AKI *via* anti-inflammatory and anti-oxidative mechanisms (Chen et al., 2019; Feng et al., 2019; Yao et al., 2019). Also, we found that DEX regulated renal autophagy, a phenomenon that was accompanied by the amelioration of oxidative stress and cell apoptosis. Pretreatment with DEX significantly increased the transcription and translation of LC3 and Beclin-1. However, DEX reduced the level of PINK protein but increased its transcription. These results showed that in the presence of DEX, damaged mitochondria were recognized by PINK1, phagocytosed to form completed autophagosomes, and degraded in lysosomes, suggesting that autophagy had been restored (Parzych and Klionsky, 2014; Bingol and Sheng, 2016). To confirm that in LPS-induced AKI, the renoprotection of DEX is associated with enhanced autophagy, we used 3-MA to inhibit the effects of DEX on autophagy. 3-MA, a PtdIns3K inhibitor, is the most widely used autophagy inhibitor. After treatment with 3-MA, the increased production of ROS and activation of caspase-3 were observed, which indicated the renoprotection of DEX was reversed, and kidney damage was aggravated in LPS-induced AKI. The accumulation of PINK1 and p62 and the reduction of Beclin-1 and LC3 II were similar to the LPS group. These changes indicate that DEX enhances autophagy to reduce oxidative stress and apoptosis. Our data confirm that the inhibition of autophagy is one of the important pathogenesis mechanisms of LPS-induced AKI and that DEX enhances autophagy to alleviate kidney injury.

We also investigated the molecular mechanisms by which DEX modulates renal autophagy in LPS-induced AKI. When activated, the kinase mTOR inhibits autophagy initiation (Munson and Ganley, 2015). The phosphorylation of mTOR was increased in LPS-induced AKI, and the upregulation of p-PI3K and p-AKT were also observed. Some studies have confirmed that during the severe stages of sepsis, beneficial autophagic activities are inhibited by mTOR activation (Sun et al., 2018). Therefore, the activation of the PI3K/AKT/mTOR pathway inhibits autophagy in LPS-induced AKI, whereas pretreatment with DEX reduced the phosphorylation of PI3K, AKT, and mTOR. The application of Atip, an α_2_-AR antagonist, inhibits the effect of DEX on the phosphorylation of PI3K, AKT, and mTOR. These data suggest that DEX can inhibit the PI3K/AKT/mTOR pathway to activate autophagy through α_2_-AR stimulation. However, our study did not determine how α_2_-AR stimulation by DEX inhibits PI3K and downstream signaling and processes, and there are few reported studies on this mechanism. We will address this issue in future research.

**Figure 9:**
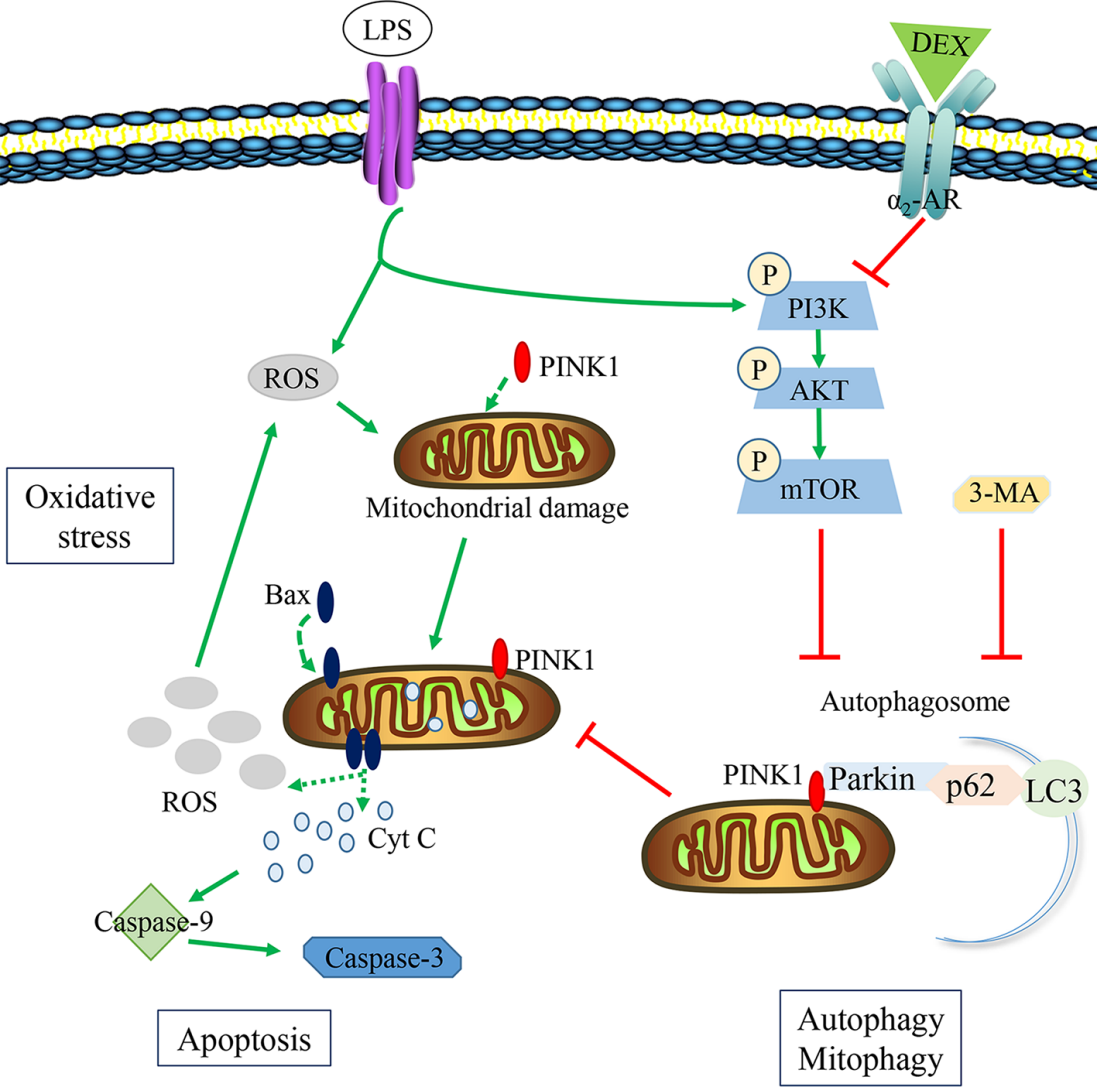
The potential protective mechanisms of DEX in reducing oxidative stress and apoptosis in LPS-induced AKI. LPS could hold back the formation of autophagosome *via* activating the PI3K/AKT/mTOR pathway, inducing the accumulation of damaged mitochondria. Then ROS and Cyt C released from damaged mitochondria could induce oxidative stress and apoptosis. DEX could inhibit the PI3K/AKT/mTOR pathway to enhance autophagy by α_2_-AR, reducing oxidative stress and apoptosis. 3-MA could inhibit the effect of DEX on autophagy.

In summary, this study provided substantial evidence for a novel mechanism in which DEX protects against LPS-induced AKI (summarized in **Figure 9**). LPS inhibited the formation of autophagosomes by activating mTOR, which subsequently inhibited autophagy. This resulted in increased oxidative stress and apoptosis, leading to kidney damage. The inhibition of autophagy is one of the vital pathogenesis of LPS-induced AKI. DEX inhibited the PI3K/AKT/mTOR pathway *via* the α_2_-AR and restored autophagy. The enhancement of autophagy removed damaged mitochondria and reduced oxidative stress and apoptosis in LPS-induced AKI.

## Data Availability Statement

The datasets generated for this study are available on request to the corresponding author.

## Ethics Statement

The animal study was reviewed and approved by the Animal Experimental Committee of Northeast Agricultural University.

## Author Contributions

HF and YZ contributed conception and design of the study. YZ, TY, and HC conducted experiments. YZ organized the database. JS and CW performed the statistical analysis. YZ wrote the first draft of the manuscript. XF and BL wrote sections of the manuscript. All authors contributed to manuscript revision, read and approved the submitted version.

## Funding

This work was supported by the National Natural Science Foundation of China (Grant Nos. 31772806 and 31802251) and the National Key Research and Development Project of China (Grant No. 2016YFD0501008).

## Conflict of Interest

The authors declare that the research was conducted in the absence of any commercial or financial relationships that could be construed as a potential conflict of interest.
